# Generalized Lymph Node Activation after Influenza Vaccination on ^18^F FDG-PET/CT Imaging, an Important Pitfall in PET Interpretation

**DOI:** 10.22038/aojnmb.2017.8702

**Published:** 2017

**Authors:** Narjess Ayati, Sarah Jesudason, Salvatore U. Berlangieri, Andrew M. Scott

**Affiliations:** 1 Nuclear medicine Research Centre, Mashhad University of Medical Sciences, Mashhad, Iran; 2 Department of Molecular Imaging & Therapy, Austin Health, Heidelberg, Victoria, Australia; 3 Olivia Newton-John Cancer Research Institute; and School of Cancer Medicine, La Trobe University, Australia; 4 Department of Medicine, University of Melbourne, Austin Health, Heidelberg, Victoria, Australia

**Keywords:** ^18^F FDG-PET, False positive, Immunization, Influenza vaccination, Lymph node

## Abstract

We report on a 59-year-old female patient with an infected vascular graft investigated with ^18^F FDG-PET/CT. The first of two studies showed FDG activity in the left deltoid and ipsilateral axillary lymph nodes explained by influenza vaccination the day prior. The second ^18^F FDG-PET/CT showed multiple FDG-avid lymph nodes on both sides of the diaphragm without tracer accumulation at the vaccination site. Three months later the CT was negative for lymphadenopathy within the chest or abdominal region. Although influenza vaccination is a potential source of false positive results in FDG PET studies, generalised lymph node activation post vaccination is a rare finding with only one prior published report in individuals infected with HIV-1. This case emphasizes the necessity of taking a history of vaccination prior to a FDG PET study, and consideration of a vaccine-related immune response even without evidence of tracer activity at the vaccination site when generalised FDG-avid lymphadenopathy is encountered.

## Introduction

Influenza vaccination is a potential source of false positive results in FDG PET studies (1-7) as 80% of patients with a history of vaccination less than 7 days before imaging, showed ipsilateral radiotracer accumulation in axillary lymph nodes (8). The aim of this report is to alert the imaging community to potential false positive findings related to current immunization programmes against H1N1 influenza virus.

## Case report

A 59 year old female patient presenting with two weeks of lower back pain, nausea, fatigue and chills was referred for investigation of an infected aorto-bifemoral graft. The CT component of the PET/CT study showed fat stranding and collections around the graft. The FDG component demonstrated diffuse metabolic activity surrounding the aortic graft and multiple FDG-avid foci indicating an infected graft with multiple abscesses. The FDG PET study also showed activity in the left deltoid and left axillary lymph nodes. (A) On questioning, the patient gave a history of having had the influenza vaccination the day prior.

Intravenous antibiotics were started and 26 days later the patient was referred for a repeat FDG PET/CT study to assess treatment response. The PET/CT study showed reduced but persistent FDG activity around the aortic graft indicating an incomplete metabolic response to antibiotic therapy. In addition, the FDG PET/CT study demonstrated enlarged lymph nodes with FDG accumulation on both sides of the diaphragm involving the bilateral cervical and axillary, porta hepatis, coeliac and left femoral nodal stations. Interestingly, the previously documented FDG uptake in the left deltoid muscle related to the influenza vaccination had completely resolved (B). Three months after removal of the infected graft and femoral vein reconstruction, the patient was assessed by CT angiogram revealing resolution of the lymphadenopathy above or below diaphragm (C).

## Discussion

Generalized lymph node activation following influenza vaccination is a rare and unexpected finding with only one prior published report in HIV-1-infected individuals (9) while abdominal lymph node activation post immunization has not been previously described. Immunization not only can result in FDG accumulation in ipsilateral and less commonly contralateral (10) axillary lymph nodes but it can also cause generalized lymph node activity, a pitfall for misinterpretation and consequently, unnecessary biopsy or patient over-treatment. In our patient, the first PET/CT study identified FDG uptake in the left deltoid muscle and left axillary nodes (A); however, on the second PET/CT study, the activity at the vaccination site and in the left axillary nodes had resolved. Although the pattern and duration of lymph node activation post immunization is not clearly understood, previous data shows variability in distribution, intensity and time to resolution of this transient inflammatory reaction (11). A retrospective study which assessed the FDG PET/CT pattern after immunization reported no abnormal tracer accumulation in any nodal region after 7 days post vaccination (8) while another study reported persistency of nodal activity up to 1 month after vaccination (10). Both mentioned papers report a variable number of active lymph nodes among examinees. This report describes another differing distribution of nodal activity following immunization. Unfortunately, pathological confirmation of the nature of the lymphadenopathy is not available in this patient; however, follow up of the patient for 12 months, including the diagnostic CT did not show lymphadenopathy within the chest or abdominal region. This case emphasizes the need of taking a history of vaccination before any FDG PET study even without evidence of tracer activity in the vaccination site.

**Figure 1:**
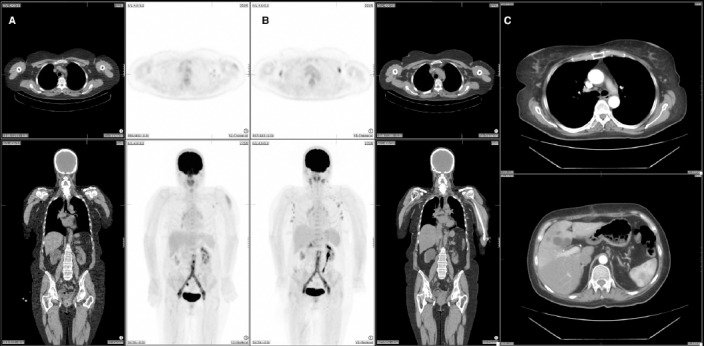
The first FDG-PET/CT study shows activity in the left deltoid and ipsilateral axillary lymph nodes (A). The second PET/CT study shows less but persistent FDG activity around the aortic graft as well as multiple FDG-avid lymph nodes on both sides of the diaphragm including bilateral cervical, bilateral axillae, porta hepatis, coeliac and left femoral regions (B). CT angiogram three months after removal of the infected graft, revealed no lymphadenopathy above or below diaphragm (C).

## Conflict of interest

The authors declare no conflict of interest.
